# Proximity ligation assay reveals both pre- and postsynaptic localization of the APP-processing enzymes ADAM10 and BACE1 in rat and human adult brain

**DOI:** 10.1186/s12868-020-0554-0

**Published:** 2020-02-04

**Authors:** Jolanta L. Lundgren, Lina Vandermeulen, Anna Sandebring-Matton, Saheeb Ahmed, Bengt Winblad, Monica Di Luca, Lars O. Tjernberg, Elena Marcello, Susanne Frykman

**Affiliations:** 1 Department of Neurobiology, Care Sciences and Society, Center for Alzheimer Research, Division of Neurogeriatrics, Karolinska Institutet, BioClinicum J9:20, Visionsgatan 4, 171 64 Solna, Sweden; 2 Department of Pharmacological and Biomolecular Sciences, Universitá Degli Studi Di Milano, Milan, Italy; 3 Department of Diagnostic and Interventional Radiology, University Medical Center, Göttingen, Germany

**Keywords:** Alzheimer disease, Amyloid precursor protein, Secretases, Synapse, Nerve terminal

## Abstract

**Background:**

Synaptic degeneration and accumulation of amyloid β-peptides (Aβ) are hallmarks of the Alzheimer diseased brain. Aβ is synaptotoxic and produced by sequential cleavage of the amyloid precursor protein (APP) by the β-secretase BACE1 and by γ-secretase. If APP is instead cleaved by the α-secretase ADAM10, Aβ will not be generated. Although BACE1 is considered to be a presynaptic protein and ADAM10 has been reported to mainly localize to the postsynaptic density, we have previously shown that both ADAM10 and BACE1 are highly enriched in synaptic vesicles of rat brain and mouse primary hippocampal neurons.

**Results:**

Here, using brightfield proximity ligation assay, we expanded our previous result in primary neurons and investigated the in situ synaptic localization of ADAM10 and BACE1 in rat and human adult brain using both pre- and postsynaptic markers. We found that ADAM10 and BACE1 were in close proximity with both the presynaptic marker synaptophysin and the postsynaptic marker PSD-95. The substrate APP was also detected both pre- and postsynaptically. Subcellular fractionation confirmed that ADAM10 and BACE1 are enriched to a similar degree in synaptic vesicles and as well as in the postsynaptic density.

**Conclusions:**

We show that the α-secretase ADAM10 and the β-secretase BACE1 are located in both the pre- and postsynaptic compartments in intact brain sections. These findings increase our understanding of the regulation of APP processing, thereby facilitating development of more specific treatment strategies.

## Background

Alzheimer disease (AD) is a slowly progressive neurodegenerative disorder leading to impairment in memory and other cognitive functions [1]. In the Alzheimer diseased brain, abnormal aggregation of the amyloid β-peptide (Aβ) leads to the formation of senile plaques. Together with neurofibrillary tangles of hyperphosphorylated tau, these plaques form the characteristic pathological hallmarks of AD [2]. Aβ has also been shown to cause synaptic dysfunction and degeneration [3–5], which are among the earliest pathological events during the course of the disease and correlate well with cognitive decline [1, 6]. The synaptotoxicity is particularly prominent for oligomeric forms of Aβ and might affect neuronal function long before the appearance of plaques [2, 7].

Aβ is produced from the amyloid precursor protein (APP) by sequential cleavage performed by β- and γ-secretase. Non-amyloidogenic cleavage of APP by α-secretase instead of β-secretase precludes Aβ formation since the α-secretase cleavage site of APP is located within the Aβ sequence [2]. The β-site APP cleaving enzyme 1 (BACE1) is the main β-secretase of neurons [8–12] while α-cleavage mainly is performed by a disintegrin and metalloproteinase 10 (ADAM10) [13, 14]. BACE1 levels and activity are increased in AD brain compared to control [15] as well as in the brains of transgenic mice models of AD, where it accumulates around amyloid plaques and in dystrophic neurites [16, 17]. In addition, a decrease in ADAM10 activity has been shown in AD patients when compared to control subjects [18].

An important issue for our understanding of the physiological and pathological role of Aβ is to elucidate how its production is regulated. A potential regulatory mechanism for Aβ production is the subcellular localization of APP and its secretases. Knowledge about the subcellular localization can open up for therapeutic opportunities by targeting inhibitors to specific subcellular sites or by manipulating the trafficking of the proteins. The importance of protein and vesicle trafficking in AD is emphasized by the fact that risk genes for AD, identified in genome wide association studies, are involved in these processes [19].

BACE1 is active in acidic environments, mainly in endosomes, and is generally considered to be located presynaptically in neurons [17, 20–23]. ADAM10, on the other hand, is mainly regarded to be localized to Golgi and the plasma membrane and to be part of the postsynaptic density (PSD) [24–26]. Based on these earlier studies, the amyloidogenic processing thus appears to take place presynaptically whereas the non-amyloidogenic processing would be considered to occur postsynaptically. However, using subcellular fractionation of rat brain and proximity ligation assay (PLA) in primary neurons, we have previously shown that both ADAM10 and BACE1 are highly enriched in synaptic vesicles (SVs). Aβ can also be produced and stored in SVs, although we could not detect any enrichment of Aβ production in pure synaptic vesicles [27–29]. However, since primary neurons do not recapitulate the situation in adult brain and due to the fact that homogenization and subcellular fractionation can introduce artefacts, it would also be important to study the localization of ADAM10 and BACE1 in sections from intact adult brain. In addition, not only the presynaptic but also the postsynaptic localization of these enzymes should be investigated. Furthermore, no studies have, to our knowledge, investigated the localization of ADAM10 and BACE1 in human brain. Normal immunohistochemistry can never reach a spatial resolution less than 200 nm and can therefore not provide detailed information about subcellular localization. In addition, novel methods such as super-resolution microscopy are not yet fully developed to be used in brain tissue and are hampered by the extensive auto-fluorescence in *postmortem* aged human brain. Therefore, we used brightfield proximity ligation (PLA) as an alternative approach to investigate the proximity of ADAM10 and BACE1, as well as their substrate APP, to the presynaptic marker synaptophysin and the postsynaptic marker PSD-95. In PLA, secondary antibodies are conjugated to oligonucleotides that, if the proteins of interest are within 40 nm distance from each other, can ligate to each other and be amplified and visualized [30]. The close proximity required thus provides much more detailed information than conventional immunohistochemistry. Using this method, as well as subcellular fractionation, we found that ADAM10 and BACE1 are located both pre- and postsynaptically in the adult rat brain as well as in human brain and that the distribution of the enzymes appears to be similar. Furthermore, we detected close proximity of APP with ADAM10, BACE1, synaptophysin and PSD95, suggesting that APP can be cleaved by ADAM10 and BACE1 both pre- and postsynaptically.

## Results

In this study, we took advantage of the highly sensitive method PLA to visualize the in situ localization of ADAM10 and BACE1 in intact adult rat and human brain. With brightfield PLA, two proteins in close proximity (< 40 nm) can be visualized in situ*.* Thus, this method provides much more detailed information than normal immunohistochemistry and also circumvents the problem of auto-fluorescence, which is particularly prominent in aged human brain. We performed all PLA experiments in both hippocampal and cortical sections of rat and human brain, but since the results were similar, we have chosen only to present the data from the hippocampal sections.

### Detecting pre- and postsynaptic ADAM10 and BACE1 in adult rat brain

**Fig. 1:**
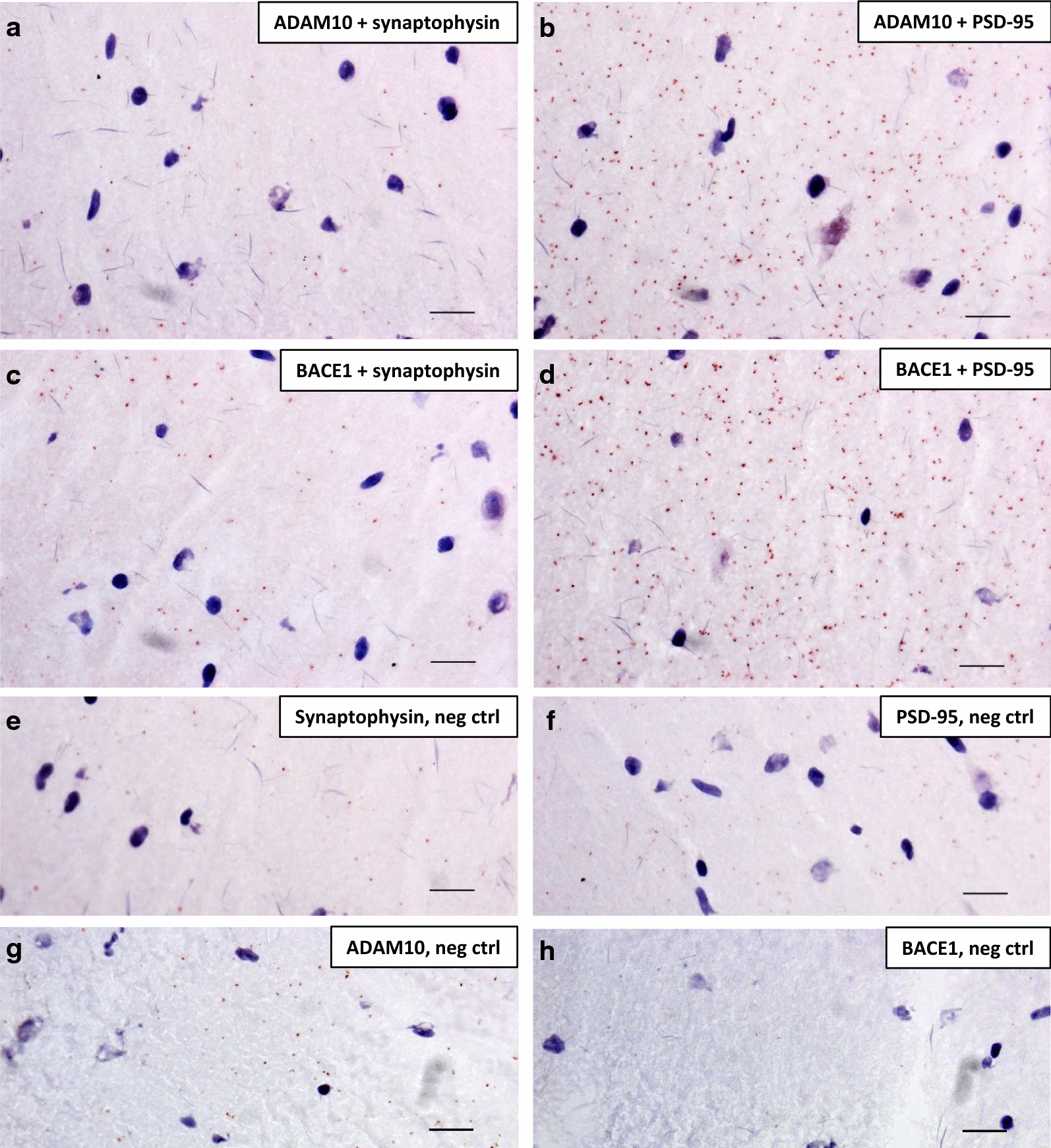
Proximity ligation assay showing pre- and postsynaptic localization of ADAM10 and BACE1 in adult rat brain. Adult rat brain hippocampal sections were labelled with primary antibodies (anti-ADAM10 or anti-BACE1 made in rabbit and anti-synaptophysin or anti-PSD-95 made in mouse) followed by secondary oligonucleotide-linked probes. Each PLA signal (brown dot) denotes two proteins within 40 nm distance from each other; **a** ADAM10 and synaptophysin, **b** ADAM10 and PSD-95, **c** BACE1 and synaptophysin and **d** BACE1 and PSD-95. In the negative control samples only one primary antibody (anti-synaptophysin (**e**), anti-PSD-95 (**f**), anti-ADAM10 (**g**) or anti-BACE1 (**h**)) was used. Brain tissue and cell nuclei were visualized by a nuclear stain solution containing Mayer´s haematoxylin. Each experiment was performed five times (technical replicates) and representative images are shown. Scale bar 20 µm

To follow up on our previous study demonstrating close proximity of ADAM10 and BACE1 to the synaptic vesicle marker synaptophysin in mouse primary hippocampal neurons [27], we here investigated the synaptic localization of ADAM10 and BACE1 in situ in thin sections of intact adult rat hippocampus. In addition to investigating the proximity of these enzymes to the presynaptic marker synaptophysin, we also investigated the proximity to the postsynaptic marker PSD-95 and whether the pre- and postsynaptic distribution differ between ADAM10 and BACE1. Using the brain from one rat, we performed PLA for the combinations ADAM10 + synaptophysin (Fig. 1a), ADAM10 + PSD-95 (Fig. 1b), BACE1 + synaptophysin (Fig. 1c) and BACE1 + PSD-95 (Fig. 1d). All these combinations gave rise to more signals compared to the negative controls where only one of the antibodies were used (Fig. 1e–g), revealing the presence of ADAM10 and BACE1 at both the pre- and postsynaptic side of the synapse in adult rat brain hippocampus.

The number of PLA signals generated with PSD-95 was generally higher than with synaptophysin for both of the secretases (Fig. 1a–g). This could indicate that both ADAM10 and BACE1 are present in higher amounts at the postsynaptic side. However, we cannot rule out that the higher number of signals is due to higher concentrations of PSD-95 than synaptophysin and/or higher affinity of the PSD-95 antibody.

From this descriptive experiment we did not observe any differences between ADAM10 and BACE1 in the number of signals detected together with synaptophysin and PSD-95. Hence, the pre- and postsynaptic distribution of ADAM10 and BACE1 in adult rat brain appears to be similar.

### Pre- and postsynaptic distribution of APP in adult rat brain

**Fig. 2:**
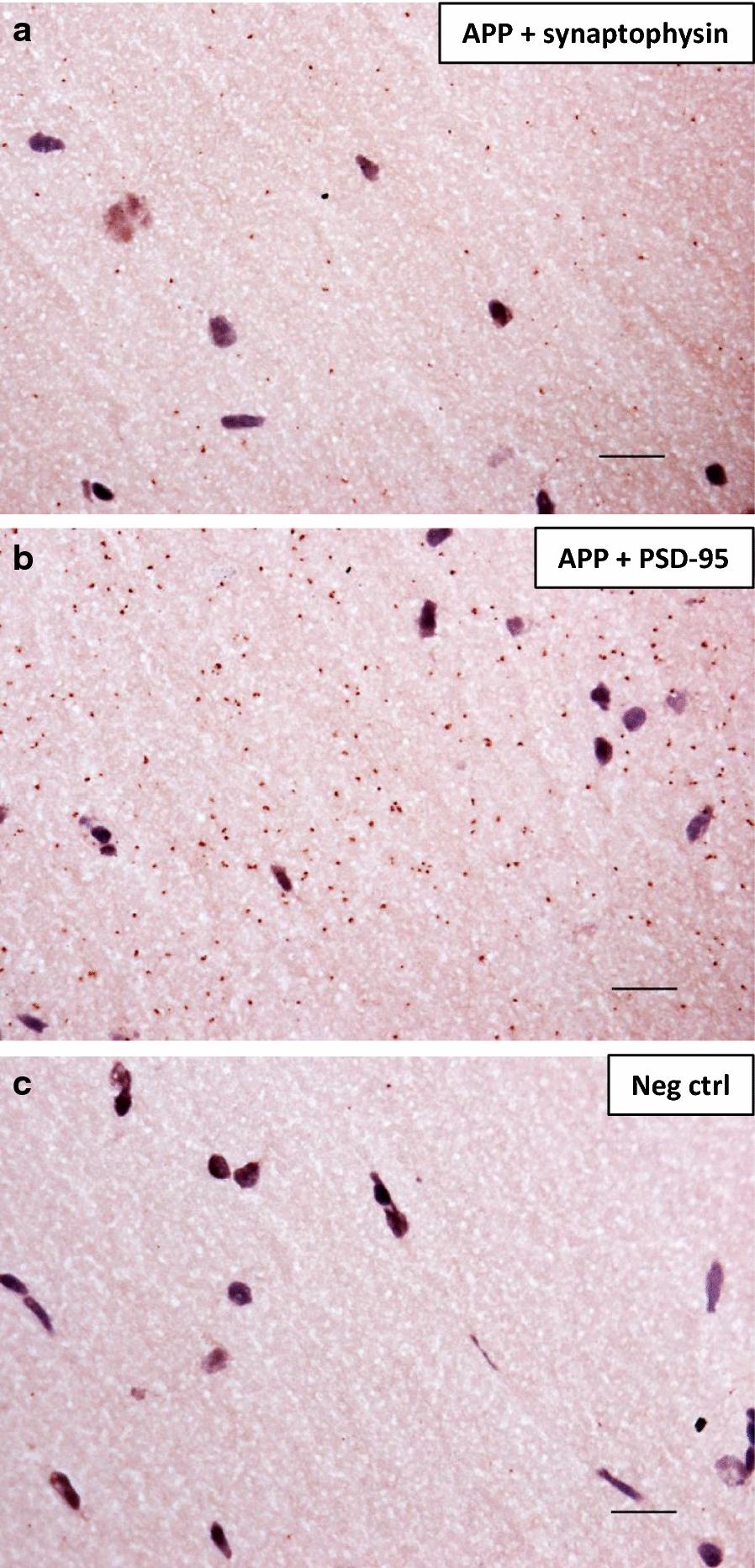
Proximity ligation assay showing pre- and postsynaptic localization of APP in adult rat brain. Adult rat brain hippocampal sections were subjected to PLA and each signal (brown dot) generated denotes two proteins within 40 nm distance from each other. **a** APP and synaptophysin, **b** APP and PSD-95. **c** Negative control with no primary antibodies. Brain tissue and cell nuclei were visualized by a nuclear stain solution containing Mayer´s haematoxylin. Each experiment was performed three times (technical replicates) and representative images are shown. Scale bar 20 µm

Apart from the localization of the secretases, we also elucidated the synaptic localization of the substrate APP using brightfield PLA. APP + synaptophysin (Fig. 2a) and APP + PSD-95 (Fig. 2b) resulted in higher number of signals than the negative control in which the primary antibodies were omitted (Fig. 2c). Thus, APP is in close proximity to both synaptophysin and PSD-95 in adult rat brain hippocampus (Fig. 2). Again, a higher number of signals was observed for PSD-95 than for synaptophysin but it is evident that APP is located both pre- and postsynaptically in rat brain. In addition, we performed brightfield PLA of ADAM10 and BACE1 with their substrate APP and, as expected, concluded that both secretases are in close proximity to APP in adult rat brain hippocampus (Additional file 1: Figure S1).

### Pre- and postsynaptic localization of ADAM10 and BACE1 in fractionated rat brain

**Fig. 3:**
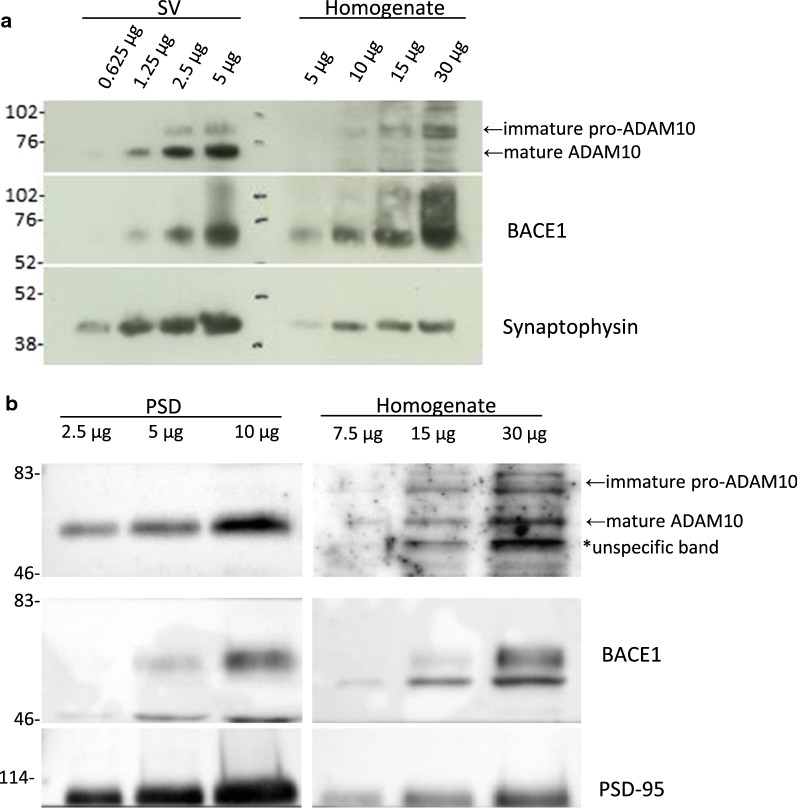
Detection of ADAM10 and BACE1 in synaptic vesicle and postsynaptic density fractions of adult rat brain. Indicated amounts of total brain homogenate (H), synaptic vesicle (SV) (**a**) and postsynaptic density (PSD) (**b**) fractions were loaded on a gel and subjected to western blotting using ADAM10, BACE1, and synaptophysin (**a**) or PSD-95 (**b**) antibodies. The SV fractionation was performed four times (from different pools of 20 rat brains) and a representative image is shown. The PSD fractionation was only performed once (from a pool of 30 rat brains)

To corroborate our PLA results, we isolated pure SV and PSD fractions from adult rat brain and used western blotting to assess the presence and potential enrichment of ADAM10 and BACE1 in these fractions. The purity of these fractions (e.g. absence of postsynaptic elements in the SV fraction and vice versa) has been shown previously [26, 27, 31]. To quantify the amounts of ADAM10 and BACE1, we loaded different concentrations of the fractions on an SDS-PAGE gel to make standard curves from which to calculate the relative amounts of the secretases. In agreement with our previously published data [27], we observed enrichment of both ADAM10 and BACE1 in the SV fractions compared to homogenate (Fig. 3a). The enrichment of mature ADAM10 (predicted size 62–64 kDa [32]) in the SV fraction compared to homogenate was on average 95-fold (n = 4 different SV preparations, individual data: 200-fold, 110-fold, 42-fold and 27-fold) and the enrichment of BACE1 was on average 24-fold (n = 4 different SV preparations, individual data: 43-fold, 27-fold, 16-fold and ninefold) but varied considerably between preparations. In accordance with our above mentioned publication [27], we also observed a similar enrichment of APP C-terminal fragments (CTFs) in the SV fractions, while full length APP was less enriched (data not shown). Interestingly, only the mature form of ADAM10 was detected in the PSD fraction.

A qualitative analysis of the enrichment of ADAM10 and BACE1 in the PSD fractions was carried out where different concentrations of homogenate and PSD fraction were loaded on a SDS-PAGE. The results show that both enzymes are present in the postsynaptic fraction and that they are enriched to a similar degree (Fig. 3b).

### Pre- and postsynaptic distribution of ADAM10 and BACE1 in human brain

**Fig. 4:**
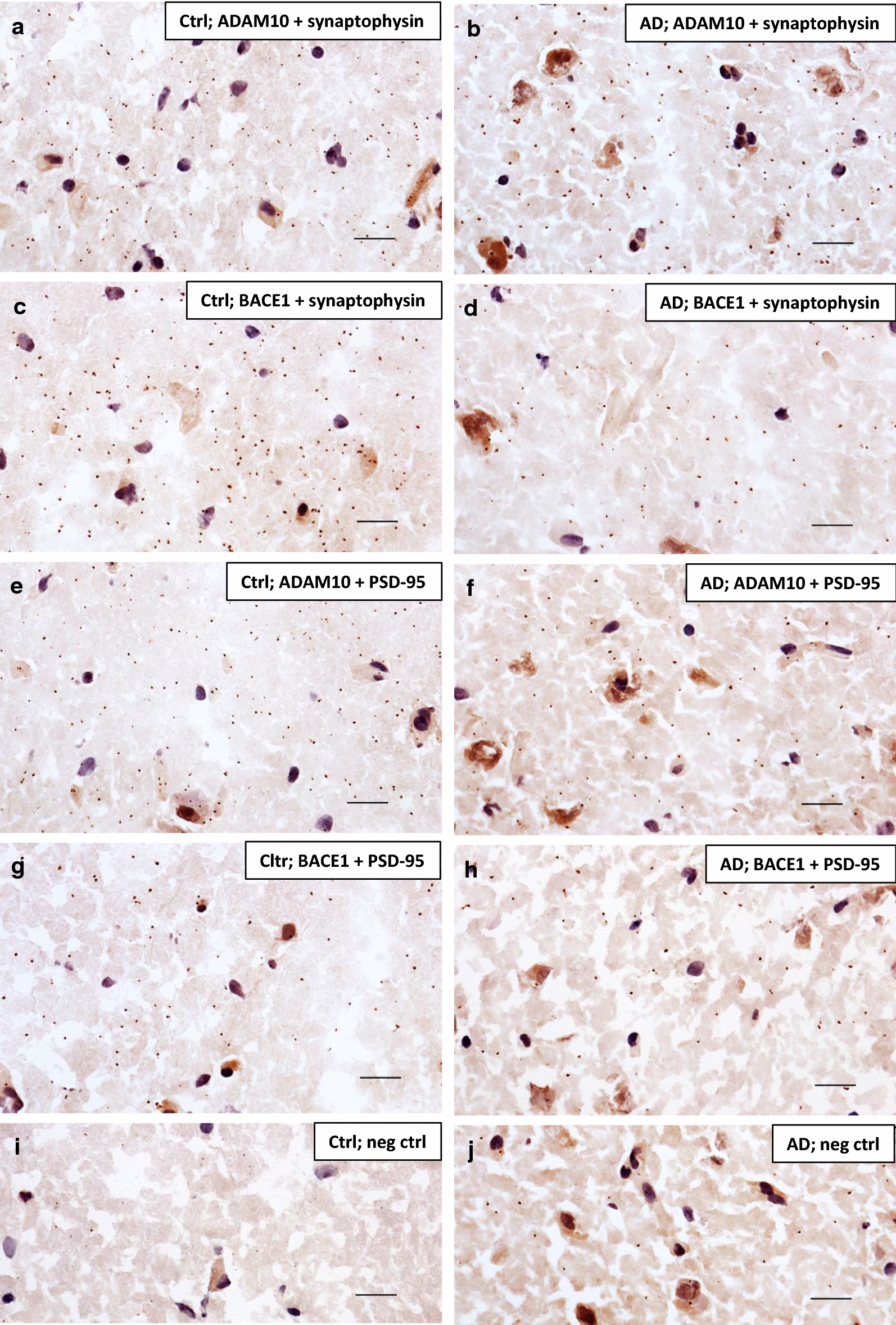
Proximity ligation assay showing pre- and postsynaptic localization of ADAM10 and BACE1 in human *postmortem* AD and control brain hippocampus. Human control (ctrl) (**a**, **c**, **e**, **g**, **i**) and AD (**b**, **d**, **f**, **h**, **j**) hippocampal sections were subjected to PLA and each signal (brown dot) generated denotes two proteins within 40 nm distance from each other, here ADAM10 and synaptophysin (**a**, **b**), BACE1 and synaptophysin (**c**, **d**), ADAM10 and PSD-95 (**e**, **f**) or BACE1 and PSD-95 (**g**, **h**). Primary antibodies were excluded from the negative control sections (**i**, **j**). Brain tissue and cell nuclei were visualized by a nuclear stain solution containing Mayer´s haematoxylin. Each experiment was performed at least three times (technical replicates) and representative images are shown. Scale bar 20 µm

Since humans develop AD spontaneously whereas rats and mice do not, and since substantial translational problems between mouse models and human patients have been observed during the last years, it is important to also study human brain. We therefore performed the same sets of PLA experiments in human *postmortem* brain as we did in rat brain. Many PLA signals were generated when ADAM10 and BACE1 were probed against both synaptophysin (Fig. 4a–d) and PSD-95 (Fig. 4e–h) in AD or control brain hippocampus, whereas only limited number of signals were detected in the negative control samples where the primary antibodies had been omitted (Fig. 4i–j). We repeated the experiment in cortical sections from 3 AD and 3 control brains and in all cases we detected both pre- and postsynaptic localization of both ADAM10 and BACE1 (data not shown). Due to large inter-experimental variations, we were unfortunately not able to quantify potential differences in the pre- and postsynaptic distribution of the secretases between AD and control brain. However, as for rat brain, no evident differences in the pre- and postsynaptic distribution between ADAM10 and BACE1 could be observed.

### Visualising pre- and postsynaptic APP in human brain

**Fig. 5:**
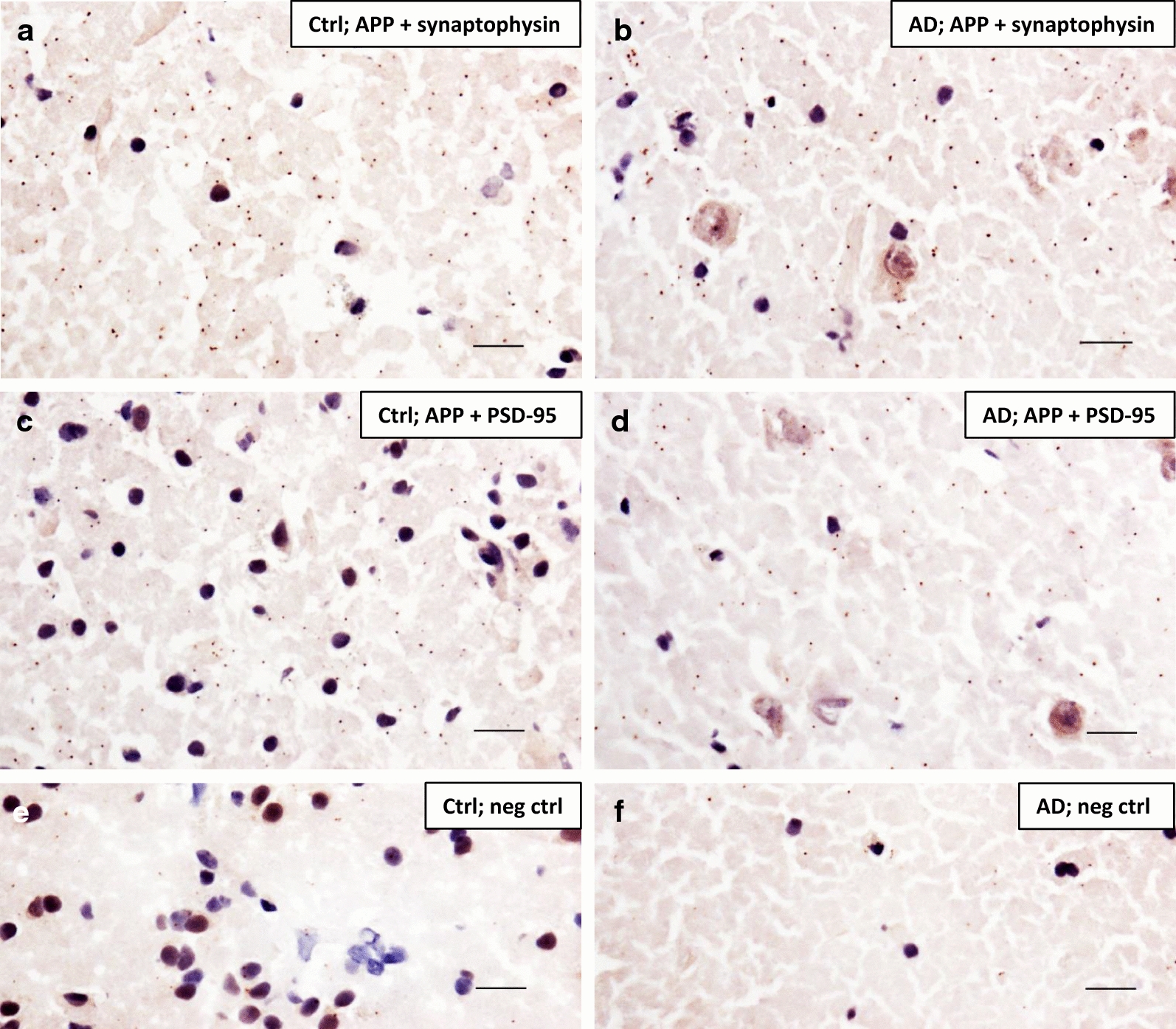
Proximity ligation assay showing pre- and postsynaptic localization of APP in human *postmortem* AD and control brain hippocampus. Human control (ctrl) (**a**, **c**) and AD (**b**, **d**) hippocampal sections were subjected to PLA and each signal (brown dot) generated denotes two proteins within 40 nm distance from each other. **a**, **b** APP and synaptophysin, **c**, **d** APP and PSD-95. Primary antibodies were excluded from the negative control (**e**, **f**) sections. Brain tissue and cell nuclei were visualized by a nuclear stain solution containing Mayer´s haematoxylin. Each experiment was performed three times (technical replicates) and representative images are shown. Scale bar 20 µm

As in rat brain (Fig. 2), we detected PLA signals when investigating the proximity of APP and synaptophysin (Fig. 5a, b) as well as APP and PSD-95 (Fig. 5c, d) also in human AD and control hippocampus. On the contrary, very few signals were detected in the control experiments where the primary antibodies were omitted (Fig. 5e, f). Again, we could unfortunately not quantify the results to compare AD to control. APP also co-localized with both ADAM10 and BACE1 in human AD and control brain hippocampus (Additional file 2: Figure S2).

## Discussion

Due to the central role of Aβ in the pathology of AD and its toxic effects on synaptic function, it is essential to understand how its production is regulated. A key regulatory step for Aβ production is the subcellular location of APP and the secretases responsible for its initial processing. Thus, elucidating the location of these secretases is of major importance. Here we show that the α-secretase ADAM10, the β-secretase BACE1 and their substrate APP are located both pre- and postsynaptically with a similar distribution pattern.

The expression of both ADAM10 and BACE1 mRNA is widespread in the human brain [33, 34] and the protein level as well as the activity of BACE1 are increased in AD compared to control brain [15]. At the synapse, BACE1 is generally thought of as a pre-synaptic enzyme [17, 20–23] while ADAM10 is known to be present in the postsynaptic membrane [26, 35]. However, we have recently demonstrated that not only BACE1, but also ADAM10, is greatly enriched in the presynaptic compartment, more specifically in synaptic vesicles [27]. We have also previously shown that both ADAM10 and BACE1 are present in postsynaptic fractions of mouse brain and that their enrichment correlates with the purity of the postsynaptic fraction [26].

Using a different fractionation protocol than ours, Pliássova et al*.* [36, 37] found most ADAM10 to be postsynaptic and most BACE1 to be localized outside of the presynaptic active zone in rodent and human brain. They also report that APP mainly is presynaptic but also postsynaptic [36, 37] and that the synaptic levels of APP were decreased during aging while ADAM10 and BACE1 levels were not affected. Since the fractionation protocols and the homogenization process can affect the results, we also used intact brain and assessed the in situ localization of the secretases and APP in the present study.

Because of the importance of the hippocampus for learning and memory and the fact that it is extensively affected by neuronal death as well as plaques and tangles in the AD brain [2, 38], we focused mainly on the hippocampus in this study although all experiments were replicated in cortical sections as well. In both of these brain regions in rat and human adult brain we observed close proximity of both ADAM10 and BACE1 to the presynaptic marker synaptophysin as well as to the postsynaptic marker PSD-95, indicating the presence of these secretases both pre- and postsynaptically. Although our study did not provide enough statistical power to allow for quantitative analysis, the proportion of pre- and postsynaptic localization appeared similar for ADAM10 and BACE1. In line with this observation, Wang et al*.* [39] reported that endogenous ADAM10 and BACE1 can physically interact with each other. Unfortunately, due to large inter-experimental variations, we were unable to quantify potential differences in the pre- and postsynaptic distribution of the secretases between AD and control brain which was our initial intention. Reasons for this could be long *postmortem* intervals and difficulties in defining exactly the same region in the different sections.

The postsynaptic localization of BACE1 is particularly interesting since some BACE1 substrates, e.g. Seizure 6 (Sez6) [40, 41] have been found postsynaptically. The importance of Sez6 for postsynaptic functions of BACE1 was demonstrated by Zhu et al*.* who used two-photon microscopy to show that dendritic spine deficits introduced by BACE1 inhibition do not arise in Sez6^−/−^ dendrites [41]. Their study also suggests that the synaptic deficits caused by BACE1 inhibitors would be of postsynaptic origin. On the other hand, ADAM10 also cleaves substrates which are located both pre- and postsynaptically as well as transsynaptically [14]. Therefore, the pre- and postsynaptic localization of both ADAM10 and BACE1 which we present evidence for in this study and in our previous studies [26, 27] is of physiological relevance.

Interestingly, in this study we show that the synaptic distribution pattern of ADAM10, BACE1 and APP seem to be similar both in rat and human brain. Our results also indicate that the proteins required for the initial step of Aβ generation are located both pre- and postsynaptically and that the balance between amyloidogenic and non-amyloidogenic processing is similar in pre- and postsynaptic compartments. Yet, for Aβ to be generated, APP is cleaved not only by BACE1 but also by γ-secretase. Previous work in our laboratory using super-resolution microscopy have demonstrated both pre- and postsynaptic localization of active γ-secretase [42]. However, the exact location of γ-secretase might be different from that of ADAM10 and BACE1 since the components of the γ-secretase complex are not enriched in SVs compared to total brain homogenate [27]. Moreover, only few PLA signals were generated when we probed for the γ-secretase complex and synaptophysin in primary neurons [27]. Nevertheless, a recent publication from our group showed, by using super-resolution microscopy, that a significant proportion of Aβ42 itself overlapped with the synaptic vesicle marker synaptophysin although other pre-synaptic structures might also contain Aβ [29].

The AD field is in urgent need of new and better therapies [43]. Reducing Aβ production by selectively inhibiting BACE1 cleavage of APP, and not of other substrates, is an attractive strategy. This can, for example, be achieved by targeting inhibitors to subcellular locations with enriched levels of both BACE1 and APP. Likewise, increasing ADAM10 cleavage of APP, which consequently would reduce Aβ production, could also be an interesting approach. This and other studies investigating the subcellular and synaptic distribution of the APP cleaving secretases are therefore vital for the development of such approaches and consequently for the advancement of AD therapy research.

## Conclusions

We conclude that both ADAM10 and BACE1 as well as APP, can be found on both the pre- and postsynaptic side of the synapse and that the distribution between these locations are similar for ADAM10 and BACE1. Although descriptive and not quantitative, the findings presented in this study are of great relevance for the understanding of synaptic APP processing and hence for the development of treatment strategies for AD.

## Methods

### Postmortem material

The use of human brain material in this study was conformed to the Declaration of Helsinki and approved by the regional ethical review board of Stockholm (2015/1803-31/2). Fresh frozen brain tissue from hippocampus of one AD and one control subject and cortex of three AD and three control subjects was obtained from the Harvard Brain Tissue Resource Center at the NIH Brain Bank, US. All subjects were female, 71 to 81 years of age, and the tissues were collected with *postmortem* delays of 8 to 27 h. The AD subjects fulfilled Braak stage V and VI while the control subjects were Braak stage I and II.

### Animals

All animals used in this study were handled according to the Swedish, German or Italian national guidelines and the guidelines of Karolinska Institutet, the University of Göttingen and the University of Milano as well as the current European Law (Directive 2010/63/EU). Animals were maintained on a 12-h light/dark cycle in a temperature-controlled room (20–22 °C) in spacious cages with free access to food and water. Tubes were provided as hideouts and paper tissues were available for the rats’ occupation and nest building. The male Wistar rats (Charles River) used for PLA were directly sacrificed by increasing levels of carbon dioxide and were instantly decapitated. The Sprague–Dawley rats (Charles River) used for the SV-fractionation were sacrificed by cervical dislocation followed by decapitation while the Sprague–Dawley rats (Charles River) used for the PSD-fractionation were anaesthetized with isoflurane (3–4%, inhalation for 2–3 min) before they were sacrificed by carbon dioxide and decapitated. The use of rat brain in this study was approved by the Animal research ethical committee of southern Stockholm (S21-14) and the Italian Ministry of Health (#326/2015). No experiments were performed on live animals. For PLA, the brains were dissected out and immediately snap frozen in liquid nitrogen or isopentane precooled to − 80 °C. A Leica CM 3050 cryostat was used to cut 10 µm thin brain sections which were mounted on Superfrost™ Plus Gold slides (Thermo Fisher Scientific) and stored at − 20 °C until use.

### Antibodies and reagents

Primary antibodies for PLA and western blotting are described in Table 1. Secondary antibodies (horseradish peroxidase (HRP)-Goat anti-mouse and HRP-Goat anti-rabbit) were purchased from Bio-Rad and GE Healthcare. Secondary antibodies conjugated to PLA probes were purchased from Sigma-Aldrich (Merck).

**Table 1:** List of antibodies used for western blotting (WB) and proximity ligation assay (PLA)

Antibody name	Company	Dilution (WB)	Dilution (PLA)
Anti-ADAM10	Abcam (ab39153)	1:4000	1:4000
Anti-Amyloid beta precursor protein C-terminus [Y188]	Abcam (ab32136)	1:5000	1:5000
Anti-APP C-terminus [C1/6.1]	BioLegend (802801)	–	1:1000
Anti-BACE1	Cell Signaling Technology (D10E5)	1:1000	1:200
Anti-PSD-95	Abcam (ab2723)	–	1:20 000
Anti-PSD-95	Neuromab, k28/43 (75-028)	1:1000	–
Anti-Synaptophysin	Enzo (VAM-SV011F)	1:10 000	1:3000

### Proximity ligation assay

Brightfield PLA was essentially performed according to the protocol of the manufacturer (Sigma-Aldrich). Brain sections from Wistar rats or *postmortem* human tissue were fixed for 10 min at room temperature in 4% paraformaldehyde, washed with phosphate buffered saline (PBS) and permeabilized with 0.4% CHAPSO for another 10 min at room temperature. Endogenous peroxidase activity was blocked with hydrogen peroxide solution by 10 min incubation at room temperature and the slices were then washed in washing solution A. Unspecific binding sites for antibodies were blocked for 30 min with blocking solution at 37 °C and the slices were subsequently incubated with primary antibodies overnight at 4 °C. The following day, secondary probes attached to oligonucleotides were added and, after washing, the oligonucleotides of the bound probes where ligated, amplified and visualized by addition of the detection reagent and substrate solution. After addition of the nuclear stain solution, containing Mayer´s haematoxylin, the brain sections where dehydrated in ascending concentrations of ethanol and finally xylene and subsequently mounted. The PLA signals were detected using a light microscope (Nikon Eclipse E800M) and manually counted using the Image J software. As negative controls for the PLA experiments in Fig. 1, we omitted one of the primary antibodies. However, we found that similar amounts of signals appear in the negative controls when performing PLA without any primary antibodies. Hence, as negative control for the rest of the PLA experiments, we decided to totally exclude primary antibodies. All experiments in one group for the hippocampal experiments were performed on brain sections from the same rat, AD or control brain, but the experiments were also performed in cortical sections from several brains (three AD and three control brains).

### Preparation of pure synaptic vesicles

Pure synaptic vesicles (SVs) were prepared from Sprague–Dawley rat forebrain as described before [31, 44]. Briefly, 20 rat brains were homogenized in homogenization buffer containing 320 mM sucrose and 4 mM HEPES–KOH (pH 7.4) in a Teflon-glass homogenizer (10 strokes at 900 rpm). The resulting homogenate (H) was centrifuged for 10 min at 1000*g* to remove cell debris and pellet nuclei (P1). Supernatants (S1) were pooled and centrifuged for 15 min at 10,000 g to obtain a crude synaptosomal fraction (P2), and crude brain cytosol (S2).

Synaptosomes were osmotically lysed by adding 9 vol. of ice-cold ddH_2_O containing protease-inhibitors (Roche) and homogenized in a Teflon-glass homogenizer (3 strokes at 2000 rpm). The lysed synaptosomes were centrifuged for 20 min at 25,000*g* at 4 °C to spin down lysed synaptosomal membranes (LP1). The SV containing supernatant (LS1), containing SVs and synaptosomal cytosol, was further ultracentrifuged for 2 h at 200,000*g* to separate SVs (LP2) from the synaptic cytosolic fraction (LS2). The LP2 fraction was resuspended in 40 mM sucrose and subjected to a continuous sucrose gradient centrifugation (from 0.05 M sucrose to 0.8 M sucrose) for 4 h at 82,500*g* and 4 °C. SVs from the gradient interface were further purified using size-exclusion chromatography on a CPG-column (using controlled pore glass beads), which separates large membrane structures (Peak 1) from SVs.

### Purification of postsynaptic density

To isolate PSDs from Sprague Dawley rat hippocampi, a modification of the method of Carlin et al*.* [45] was used as described by Gardoni et al*.* [46, 47]. Homogenization of 30 rat hippocampi was carried out by 10 strokes in a Teflon-glass homogenizer in 4 volumes of 0.32 M sucrose containing 1 mM HEPES, 1 mM MgCl_2_, 1 mM NaHCO_3_, 1 mM NaF and 0.1 mM phenylmethylsulfonylfluoride (PMSF) (pH 7.4) in the presence of protease inhibitors (Complete; Roche/ Phosphatase Inhibitor Cocktail 2; Sigma-Aldrich), and centrifuged at 1000×*g* for 5 min at 4 °C. The supernatant was centrifuged at 13,800×*g* for 15 min at 4 °C to obtain a fraction containing mitochondria and synaptosomes. The pellet was resuspended in 2.4 volumes of 0.32 M sucrose containing 1 mM HEPES and Complete, overlaid on a sucrose gradient (0.85–1.0–1.2 M), and centrifuged at 82,500×*g* for 2 h at 4 °C. The fraction between 1.0 and 1.2 M sucrose was removed, diluted with an equal volume of 0.5% TritonX-100 in 0.16 M sucrose containing 1 mM HEPES and stirred at 4 °C for 15 min. This solution was centrifuged at 82,500×*g* for 45 min. The resulting pellet was resuspended in 0.32 M sucrose containing 1 mM HEPES and Complete buffer, overlaid on a sucrose gradient (1.0–1.5–2.1 M), and centrifuged at 100,000×*g* for 2 h at 4 °C. The fraction between 1.5 and 2.1 M sucrose was removed and diluted with an equal volume of 1% TritonX-100, 150 mM KCl and Complete. PSDs were collected by centrifugation at 100,000×*g* at 4 °C for 45 min and stored at − 80 °C.

### SDS-PAGE and western blotting

Different concentrations of SV or PSD preparations and matching total brain homogenate were separated by SDS-PAGE on 4–12% polyacrylamide Bis–Tris gels (SV) (Invitrogen) or 7% SDS-PAGE/Tris–Glycine gels (PSD) and transferred to PVDF (SV) or nitrocellulose membranes (PSD) (Bio-Rad). The membranes were incubated with primary antibodies (Table 1) followed by secondary antibodies coupled to HRP (GE-Healthcare or Bio-Rad). Signals were visualized with SuperSignal West Pico enhanced chemiluminescent reagent (Pierce), Immobilon Western Chemiluminescent HRP Substrate (Millipore) or Clarity™ Western ECL substrate (Bio-Rad) and exposure to film (GE-Healthcare), LAS-3000 CCD camera (Fujifilm) or computer-assisted imaging (ChemiDoc system and Image lab 4.0 software; Bio-Rad).

## Supplementary information

**Additional file 1.** Proximity ligation assay showing co-localization of ADAM10 and BACE1 with their substrate APP in adult rat brain. Adult rat brain hippocampal sections were subjected to PLA and each signal (brown dot) generated denotes two proteins within 40 nm distance from each other. (a) APP and ADAM10, (b) APP and BACE1. (c) Negative control with no primary antibodies. Brain tissue and cell nuclei were visualized by a nuclear stain solution containing Mayer´s haematoxylin. Each experiment was performed three times and representative images are shown. Scale bar 20 µm.

**Additional file 2.** Proximity ligation assay showing co-localization of ADAM0 and BACE1 with their substrate APP in human post mortem AD and control brain hippocampus. Human control (ctrl) (a, c and e) and AD (b, d and f) hippocampal sections were subjected to PLA and each signal (brown dot) generated denotes two proteins within 40 nm distance from each other. (a–b) APP and ADAM10, (c–d) APP and BACE1. Primary antibodies were excluded from the negative control (e–f) sections. Brain tissue and cell nuclei were visualized by a nuclear stain solution containing Mayer´s haematoxylin. Each experiment was performed three times and representative images are shown. Scale bar 20 µm.

## Data Availability

The datasets used and/or analysed during the current study are available from the corresponding author or from the archive at Karolinska Institutet on reasonable request.

## Abbreviations

Aβ: Amyloid β-peptide
AD: Alzheimer disease
ADAM10: A disintegrin and metalloproteinase 10
APP: Amyloid precursor protein
BACE1: β-Site APP cleaving enzyme 1
CTF: c-Terminal fragment
H: Homogenate
HRP: Horseradish peroxidase
PLA: Proximity ligation assay
PMSF: Phenylmethylsulfonylfluoride
PSD: Post-synaptic density
PVDF: Polyvinylidene difluoride
Sez6: Seizure 6
SV: Synaptic vesicle
