# Supplementary material for: Proximity ligation assay reveals both pre- and postsynaptic localization of the APP-processing enzymes ADAM10 and BACE1 in rat and human adult brain
Source: BMC Neurosci. 2020 Feb 4;21:6. doi: 10.1186/s12868-020-0554-0 (PMC7001251; doi:10.1186/s12868-020-0554-0)
Supplement: Supplementary file 1 — Additional file 1. Proximity ligation assay showing co-localization of ADAM10 and BACE1 with their substrate APP in adult rat brain. Adult rat brain hippocampal sections were subjected to PLA and each signal (brown dot) generated denotes two proteins within 40 nm distance from each other. (a) APP and ADAM10, (b) APP and BACE1. (c) Negative control with no primary antibodies. Brain tissue and cell nuclei were visualized by a nuclear stain solution containing Mayer´s haematoxylin. Each experiment was performed three times and representative images are shown. Scale bar 20 µm. [file 12868_2020_554_MOESM1_ESM.pptx]

## Slide 1
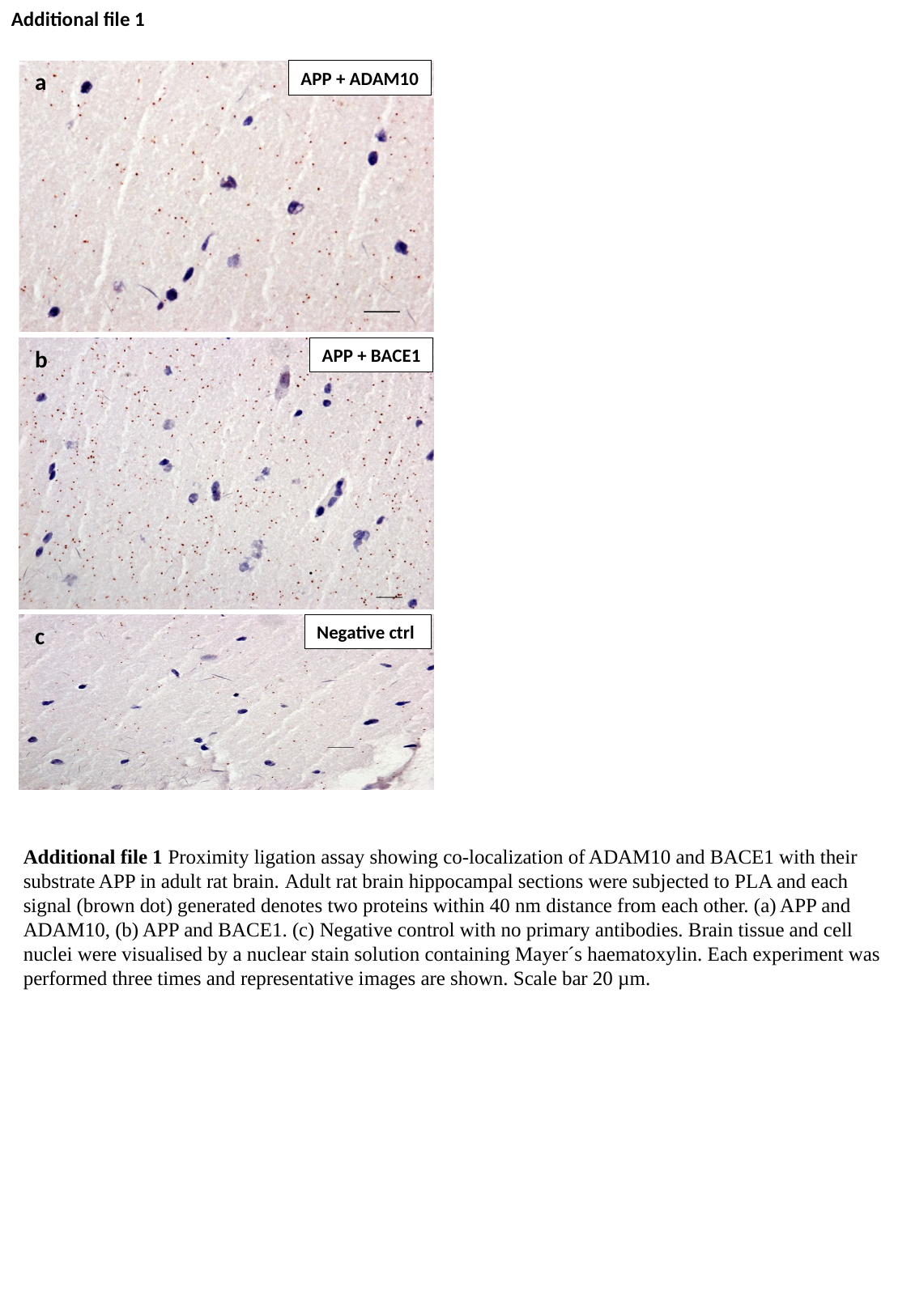

Additional file 1
a
APP + ADAM10
APP + BACE1
b
Negative ctrl
c
Additional file 1 Proximity ligation assay showing co-localization of ADAM10 and BACE1 with their substrate APP in adult rat brain. Adult rat brain hippocampal sections were subjected to PLA and each signal (brown dot) generated denotes two proteins within 40 nm distance from each other. (a) APP and ADAM10, (b) APP and BACE1. (c) Negative control with no primary antibodies. Brain tissue and cell nuclei were visualised by a nuclear stain solution containing Mayer´s haematoxylin. Each experiment was performed three times and representative images are shown. Scale bar 20 µm.
