# Supplementary material for: Proximity ligation assay reveals both pre- and postsynaptic localization of the APP-processing enzymes ADAM10 and BACE1 in rat and human adult brain
Source: BMC Neurosci. 2020 Feb 4;21:6. doi: 10.1186/s12868-020-0554-0 (PMC7001251; doi:10.1186/s12868-020-0554-0)
Supplement: Supplementary file 2 — Additional file 2. Proximity ligation assay showing co-localization of ADAM0 and BACE1 with their substrate APP in human post mortem AD and control brain hippocampus. Human control (ctrl) (a, c and e) and AD (b, d and f) hippocampal sections were subjected to PLA and each signal (brown dot) generated denotes two proteins within 40 nm distance from each other. (a–b) APP and ADAM10, (c–d) APP and BACE1. Primary antibodies were excluded from the negative control (e–f) sections. Brain tissue and cell nuclei were visualized by a nuclear stain solution containing Mayer´s haematoxylin. Each experiment was performed three times and representative images are shown. Scale bar 20 µm. [file 12868_2020_554_MOESM2_ESM.pptx]

## Slide 1
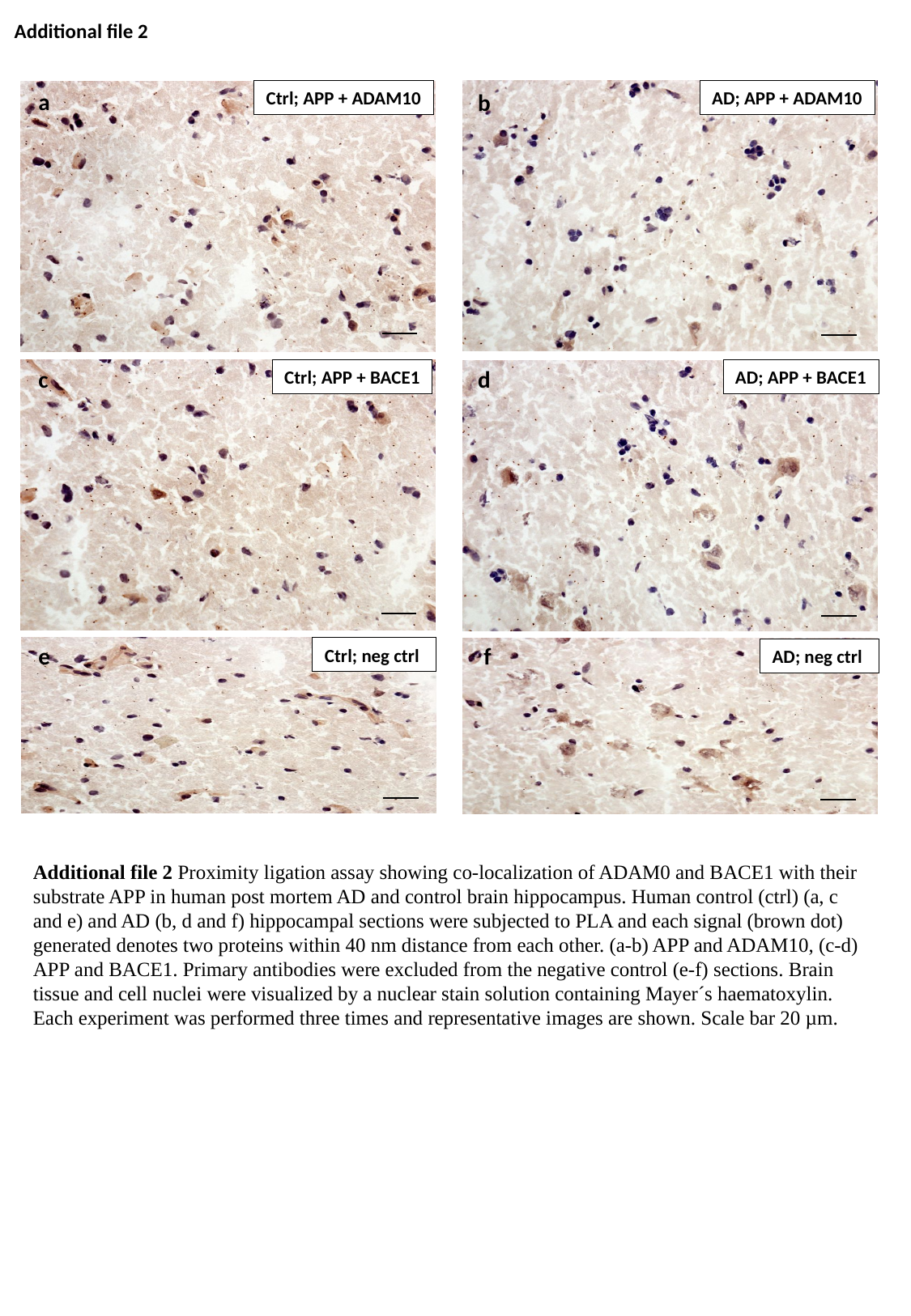

Additional file 2
Ctrl; APP + ADAM10
AD; APP + ADAM10
a
b
d
c
Ctrl; APP + BACE1
AD; APP + BACE1
e
f
Ctrl; neg ctrl
AD; neg ctrl
Additional file 2 Proximity ligation assay showing co-localization of ADAM0 and BACE1 with their substrate APP in human post mortem AD and control brain hippocampus. Human control (ctrl) (a, c and e) and AD (b, d and f) hippocampal sections were subjected to PLA and each signal (brown dot) generated denotes two proteins within 40 nm distance from each other. (a-b) APP and ADAM10, (c-d) APP and BACE1. Primary antibodies were excluded from the negative control (e-f) sections. Brain tissue and cell nuclei were visualized by a nuclear stain solution containing Mayer´s haematoxylin. Each experiment was performed three times and representative images are shown. Scale bar 20 µm.
